# Life-Threatening SARS-CoV-2–Associated Encephalopathy and Multiorgan Failure in Children, Asia and Oceania, 2022–2024

**DOI:** 10.3201/eid3202.250549

**Published:** 2026-02

**Authors:** Mariko Kasai, Hiroshi Sakuma, Motomasa Suzuki, Masahiro Nishiyama, Nanako Kawata, Jainn-Jim Lin, Kuang-Lin Lin, Velda Han, Shekeeb S. Mohammad, Russell C. Dale, Terrence Thomas, Kazuhiro Muramatsu, Osamu Mitani, Yoshiyuki Kobayashi, Kouhei Ishida, Yuichi Abe, Ichiro Kuki, Jun-ichi Takanashi

**Affiliations:** Tokyo Metropolitan Institute of Medical Science, Tokyo, Japan (M. Kasai, H. Sakuma); Aichi Children's Health and Medical Center, Aichi, Japan (M. Suzuki); Hyogo Prefectural Kobe Children’s Hospital, Kobe, Japan (M. Nishiyama); Tokyo Metropolitan Children’s Medical Center, Tokyo (N. Kawata); Chang Gung Children’s Hospital and Chang Gung Memorial Hospital, School of Medicine, College of Medicine, Chang Gung University, Taoyuan, Taiwan (J.-J. Lin, K.-L. Lin); Khoo Teck Puat-National University Children’s Medical Institute, Singapore (V. Han); Yong Loo Lin School of Medicine, National University of Singapore, Singapore (V. Han); Kids Neuroscience Centre, The Children’s Hospital at Westmead, Faculty of Medicine and Health, University of Sydney, Westmead, New South Wales, Australia (V. Han, S.S. Mohammad, R.C. Dale); KK Women’s and Children’s Hospital, Singapore (T. Thomas); Jichi Medical University, Tochigi, Japan (K. Muramatsu); Fukuyama City Hospital, Hiroshima, Japan (O. Mitani); Hiroshima University Hospital, Hiroshima (Y. Kobayashi); Sapporo Medical University School of Medicine, Sapporo, Japan (K. Ishida); National Center for Child Health and Development, Tokyo (Y. Abe); Osaka City General Hospital, Osaka, Japan (I. Kuki); Tokyo Women’s Medical University Yachiyo Medical Center, Chiba, Japan (J. Takanashi)

**Keywords:** SARS-CoV-2, COVID-19, severe acute respiratory syndrome coronavirus 2, meningitis/encephalitis, viruses, respiratory infections, brain edema, shock, multiorgan failure, cytokine release syndrome, Australia, Japan, Singapore, Taiwan

## Abstract

SARS-CoV-2 infections in children occasionally manifest with severe neurologic signs. We report a case series of life-threatening encephalopathy associated with SARS-CoV-2 in 25 children in Australia, Japan, Singapore, and Taiwan during February 2022–January 2024. All children had severe encephalopathy develop, characterized by rapidly progressive cerebral edema, conditions known as acute shock with encephalopathy and multiorgan failure or acute fulminant cerebral edema. Among the 25 patients, 22 (88%) eventually died; 11 (44%) children died within 24 hours of hospitalization. In addition, 18 (72%) had illness manifest with shock, and 14 (56%) had multiorgan failure develop within 6 hours of neurologic onset. Serum concentrations of cytokines/chemokines including interleukin 6 and tumor necrosis factor-α were significantly higher within 24 hours of onset than for controls. SARS-CoV-2–associated encephalopathy cases such as those described here represent an emerging neurologic crisis with high mortality rate resulting from rapidly progressive brain edema and multiorgan failure.

SARS-CoV-2, which primarily causes respiratory illness, is associated with diverse central and peripheral nervous system complications. Multisystem inflammatory syndrome in children is often associated with neurologic syndromes, and SARS-CoV-2–triggered encephalopathy syndromes, such as life-threatening acute fulminant cerebral edema (AFCE), have been reported (*1*,*2*). Previously, we reported that SARS-CoV-2–associated encephalopathy had a poor prognosis because of a higher incidence of AFCE and acute shock with encephalopathy and multiorgan failure (ASEM) than for non–SARS-CoV-2–associated encephalopathy (*1*,*2*). Both AFCE and ASEM are devastating, infection-triggered encephalopathy syndromes that result in high mortality rates in children (*3*) and typically occur in healthy children triggered by viral infections. Illness in such cases manifests with acute encephalopathy symptoms, shock, multiorgan failure, and rapidly progressive diffuse cerebral edema (*4*,*5*). A hypercytokine state has been suggested as the underlying mechanism for the pathogenesis of ASEM/AFCE, although reports are rare, and development of effective treatment strategies remains challenging. Several reports have addressed the emergence of severe cases of SARS-CoV-2 ASEM/AFCE in children (*6*–*8*); however, we know of no review of the literature for SARS-CoV-2 ASEM/AFCE cases, and only a few case series exist, none of which include diverse international populations.

Here, we report an international case series and a scoping review designed to outline the common clinical manifestations of SARS-CoV-2 ASEM/AFCE in diverse populations. Furthermore, we aim to describe proinflammatory cytokine and chemokine profiles in serum from patients with SARS-CoV-2 ASEM/AFCE to underscore the predominant immune pathology in the early stages of disease onset.

## Methods

### Study Population

We conducted an international multicenter collaborative study in pediatric patients <18 years of age who had ASEM/AFCE develop during or after SARS-CoV-2 infection. The study enrolled children who fulfilled the ASEM/AFCE criteria in collaborating hospitals in 4 countries—Australia, Japan, Singapore, and Taiwan—during February 2022–January 2024. We sent web-based questionnaires to child neurologists in collaborative research institutes and obtained clinical information. We collected information on major clinical symptoms, along with the timing of the symptom onset. In addition, we collected serum samples from facilities in Japan that provided consent to obtain specimens.

We used international consensus criteria (*3*,*9*) to define ASEM/AFCE as a febrile illness preceding or concurrent with the onset of neurologic manifestations, rapid reduction of consciousness, or seizures with progressive diffuse cerebral edema. Shock and multiorgan failure (>3 of the following: anemia, thrombocytopenia, disseminated intravascular coagulation, acidosis, elevated hepatocellular enzymes, and renal dysfunction) are essential for ASEM. We excluded patients who had traumatic brain injury, metabolic disorders, or marked hyponatremia. All patients tested positive for SARS-CoV-2 by reverse transcription PCR or antigen testing.

We evaluated outcomes by using a pediatric cerebral performance category (PCPC) score (*10*). In brief, we graded outcomes into 6 categories: presymptomatic state (PCPC = 1), mild disability (PCPC = 2; age-appropriate interaction, minor controlled neurologic condition), moderate disability (PCPC = 3; impaired age-appropriate functioning, uncontrolled neurologic condition), severe disability (PCPC = 4; abnormal motor responses, including no purposeful, decorticate, or decerebrate reactions to pain), coma or vegetative state (PCPC = 5), and death (PCPC = 6).

### Scoping Review

To gather additional data, we performed a scoping review, according to Preferred Reporting Items for Systematic Reviews and Meta-Analyses guidelines (*11*). In the review, we searched PubMed for articles or abstracts published during January 1, 2020–June 30, 2024. We curated English-language publications and used the following search terms: “coronavirus disease 2019 (COVID-19)” OR “SARS-CoV-2” AND (encephalitis) OR (acute fulminant cerebral edema) OR (Hemorrhagic shock and encephalopathy syndrome) OR (Acute shock and encephalopathy with multiorgan failure) NOT (autoimmune). We employed the following inclusion criteria: diagnosed cases of COVID-19, clinical courses and neuroimaging abnormalities consistent with the ASEM/AFCE diagnosis, and eligible case series, case reports, and cohort studies. Two authors (M.K. and H.S.) reviewed the articles. We standardized data extraction by using 2 categories, study characteristics (first author’s name, year of publication, country of origin, study design, and sample size) and clinical details (sex, age, medical history or comorbidities, neurologic symptoms, and radiologic findings). We excluded studies that lacked sufficient information on neurologic symptoms. A single reviewer extracted data and validated it for accuracy. We sourced the flow diagram for scoping reviews, which included searches of databases, from a previous report (*9*).

### Cytokine and Chemokine Profiles 

Of the 25 patients in the cohort, we obtained serum samples from 9 patients with ASEM/AFCE to conduct cytokine and chemokine measurement. Among the 9 patients, 2 provided 2 samples collected at different time points, resulting in a total of 11 samples analyzed. We centrifuged blood samples immediately after collection to separate serum, which we then stored at –80°C until analysis. For all samples, we recorded the precise interval from neurologic onset to blood collection and divided samples into early-stage (<24 hours after neurologic onset; n = 6) and late-stage (>24 hours after neurologic onset; n = 5). For control samples (n = 14), we used samples from patients with other inflammatory neurologic diseases who had neurologic symptoms: epilepsy (n = 1), status epilepticus (n = 6), altered mental status with seronegative autoimmune encephalitis (n = 4), abnormal behavior after influenza (n = 1), and unclassified acute encephalopathy (n = 2). All controls were children <18 years of age without neurologic autoantibodies, abnormal findings on brain imaging, or cerebrospinal fluid pleocytosis. The methods used to determine neurologic autoantibodies in the controls were reported in a previous study (*12*).

We determined serum levels of cytokines and chemokines for all samples by using the Bio-Plex suspension array system with the Bio-Plex Pro Human Chemokine Panel 11-Plex (Bio-Rad Laboratories, https://www.bio-rad.com). The assays were CXC-chemokine ligand (CXCL) 13, CXCL1, interferon-gamma (IFN-γ), interleukin (IL) 10, IL-1β, IL-6, IL-8, CXCL10, chemokine ligand 2 (CCL2), macrophage migration inhibitory factor (MIF), and tumor necrosis factor α (TNF-α). We conducted all assays in duplicate and adopted the mean values for each set of results. We diluted serum 1:4 for the assay and further diluted some serum samples to fit the calibration curve if IL-6 and IL-8 levels were beyond the detection limits. We then compared the cytokine/chemokine levels between the early-stage, late-stage, and control groups. 

#### Statistical Analysis

We evaluated cytokine and chemokine profiles by using the nonparametric Kruskal-Wallis test with post hoc Mann-Whitney U tests. We set the significance of the differences at p<0.05. We used Mann-Whitney U tests with Bonferroni adjustments (adjusted α = 0.016) for multiple testing at the cytokine/chemokine levels. We performed statistical analyses by using R software version 4.3.2 (The R Project for Statistical Computing, https://www.r-project.org).

#### Ethics and Informed Consent

The studies involving human participants were reviewed and approved by the institutional review board of Tokyo Metropolitan Institute of Medical Science, approval nos. 20-28 (*3*) and 21-2 (*6*). Written informed consent to participate in this study was provided by the participants or their legal guardian/next of kin.

## Results

### Patient Cohort

The patient cohort consisted of 25 new cases of SARS-CoV-2 ASEM/AFCE: 14 from Japan, 8 from Taiwan, 2 from Singapore, and 1 from Australia. All patients were of Asian ethnicity. We compiled complete clinical characteristics of the patients (Table 1). The age at onset ranged from 10 months to 10 years (median 2 years 11 months). The male:female ratio was 1.1:1. The medical history of 20 of the 25 patients was healthy; 4 had febrile convulsions, and 1 had previous hypoxic ischemic encephalopathy and epilepsy. All but 1 patient, whose vaccination history was unknown, had not been vaccinated against SARS-CoV-2. 

**Table 1:** Clinical characteristics of patients with SARS-CoV-2 ASEM/AFCE in study of life-threatening SARS-CoV-2–associated encephalopathy and multiorgan failure in children, Asia and Oceania, 2022–2024*

Patient no.	Age	Hours from onset of fever to neurologic symptoms	GCS at onset	Brain CT/MRI	Outcome by PCPC†	Hours from onset of neurologic symptoms	
						To RA	To CA
1	10 mo	10	E1V1M1	DCE	6	<6	>48
2	10 mo	48	NA	DCE, focal abnormality	6	NA	NA
3	1 y 6 mo	19	E4V3M4	DCE	6	NA	NA
4	1 y 10 mo	14	NA	DCE	5	>48	–
5	2 y	16	E1V1M1	DCE	6	<6	<6
6	2 y	NA	E1V1M1	DCE	6	6–12	>48
7	2 y 4 mo	5	E1V1M1	DCE	5	>48	–
8	2 y 4 mo	12	E1V1M1	DCE	6	At onset	At onset
9	2 y 7 mo	12	E1V1M3	DCE, BTA	6	NA	NA
10	2 y 7 mo	24	E1V2M1	DCE	6	<6	>48
11	2 y 8 mo	15	E1V1M3	DCE	6	6–12	>48
12	2 y 9 mo	NA	E2V2M3	DCE	6	<6	<6
13	2 y 11 mo	27	E2V1M3	DCE	6	>48	>48
14	3 y 2 mo	20	E1V2M4	DCE	6	<6	<6
15	3 y 9 mo	5~10	E1V1M4	DCE, focal abnormality	6	NA	NA
16	4 y	NA	NA	DCE	6	>48	>48
17	4 y	NA	E1V1M1	DCE	6	<6	<6
18	6 y 7 mo	10	E3V2M5	DCE	1	–	–
19	7 y 4 mo	9.5	E1V1M4	DCE	6	NA	>48
20	7 y 7 mo	18	E1V1M1	DCE	6	24–48	>48
21	8 y 1 mo	20	E1V2M1	DCE	6	24–48	24–48
22	8 y 6 mo	NA	E1V1M1	DCE	6	<6	<6
23	8 y 9 mo	NA	E2V3M4	DCE	6	<6	≥48
24	8 y 9 mo	2	E1V1M1	DCE	6	<6	<6
25	10 y 2 mo	12	NA	DCE	6	6–12	>48

*AFCE, acute fulminant cerebral edema; ASEM, acute shock with encephalopathy and multiorgan failure; BTA, bright tree appearance; CA, cardiac arrest; DCE, diffuse cerebral edema; GCS, Glasgow Coma Scale; NA, not available; PCPC, pediatric cerebral performance category; RA, respiratory arrest; –, not applicable. †Outcome was graded as follows: presymptomatic state (PCPC = 1), mild disability (PCPC = 2), moderate disability (PCPC = 3), severe disability (PCPC = 4), coma or vegetative state (PCPC = 5), and death (PCPC = 6).

**Figure 1:**
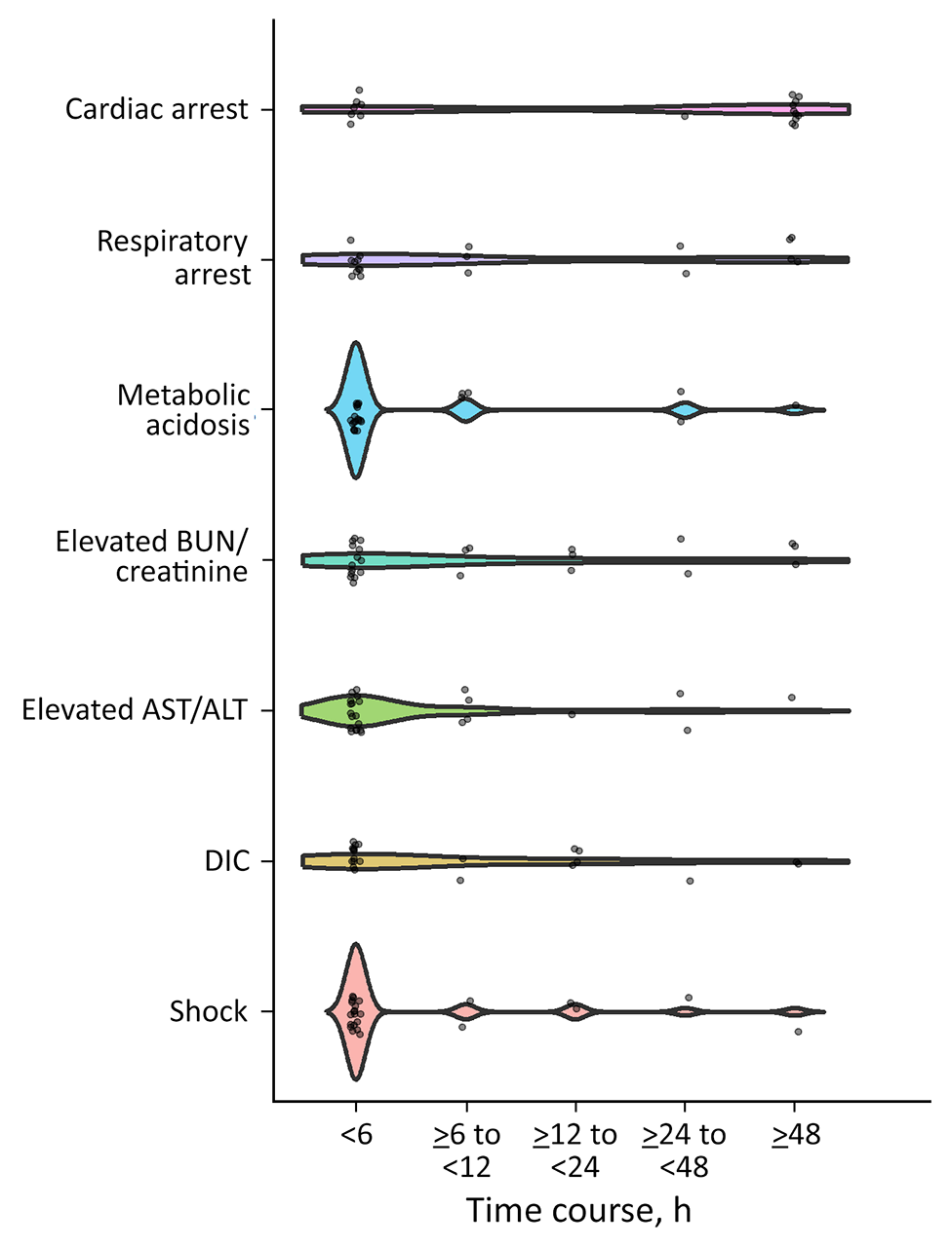
Time course of clinical manifestations of acute shock with encephalopathy and multiorgan failure/acute fulminant cerebral edema (ASEM/AFCE) for study of life-threatening SARS-CoV-2–associated encephalopathy and multiorgan failure in children, Asia and Oceania, 2022–2024. Violin plots show time from onset of neurologic symptoms; dots indicate cases and shading 95% CIs. Laboratory findings observed within 6 hours of neurologic onset in more than half of patients with SARS-CoV-2 ASEM/AFCE were as follows: increased hepatocellular enzymes (68%), elevated blood urea nitrogen or creatinine (56%), prolonged prothrombin time, partial thromboplastin time, thrombin time (52%), and metabolic acidosis (52%). ALT, alanine aminotransferase; AST, aspartate aminotransferase; BUN, blood urea nitrogen; DIC, disseminated intravascular coagulation.

None of the patients had severe respiratory symptoms before neurologic onset. Fever was evident in all patients; maximum body temperature ranged from 38.3°C to 42.4°C (median 41°C) in the acute clinical course. Neurologic symptoms appeared 2–48 (median 14.5) hours after the onset of fever. The median Glasgow Coma Scale score at initial hospital visit was 4 (range 3–11) (n = 22). Clinical seizures were evident in 23 (92%) of 25 patients in our cohort, among which status epilepticus or seizure clusters were present in 16/25 (64%) patients, whereas seizures lasting <5 minutes were present in 7 (28%) patients. Diarrhea was present in 8 (32%) of the 25 patients at the onset of neurologic symptoms. Neurologic deterioration and systemic organ damage were evident in all patients, especially in the early course of ASEM/AFCE; 18 (72%) patients had shock, 14 (56%) multiorgan failure, and 13 (52%) disseminated intravascular coagulation (DIC) within 6 hours of neurologic onset (Table 2; Figure 1). Metabolic acidosis was evident in 24 (96%) of 25 patients. 

**Table 2:** Frequency of shock and signs of multiorgan failure in patients with SARS-CoV-2 ASEM/AFCE in study of life-threatening SARS-CoV-2–associated encephalopathy and multiorgan failure in children, Asia and Oceania, 2022–2024*

Symptoms	No. (%) cases
Shock	24 (96)
DIC	22 (88)
Elevated AST or ALT†	25 (100)
Elevated BUN or creatinine	25 (100)
Thrombocytopenia‡	21 (84)
Reduced hemoglobin§	15 (60)
Metabolic acidosis	24 (96)

*ALT, alanine aminotransferase; AFCE, acute fulminant cerebral edema; ASEM, acute shock with encephalopathy and multiorgan failure; AST, aspartate aminotransferase; BUN, blood urea nitrogen; DIC, disseminated intravascular coagulation. †A total of 22 patients had AST or ALT elevations with a >4-digit range. ‡Platelet count <150,000/µL. §Hemoglobin level decreased by >3 g/dL from admission.

Immunotherapies were given to most case-patients. Intravenous methylprednisolone (30 mg/kg) was administered in 18 (72%) cases, intravenous immunoglobulin (2 g/kg) in 14 (56%) cases, and targeted temperature management (36.0°C–36.9°C) in 17 (68%) cases. Four of 25 patients were treated with intravenous tocilizumab. 

**Figure 2:**
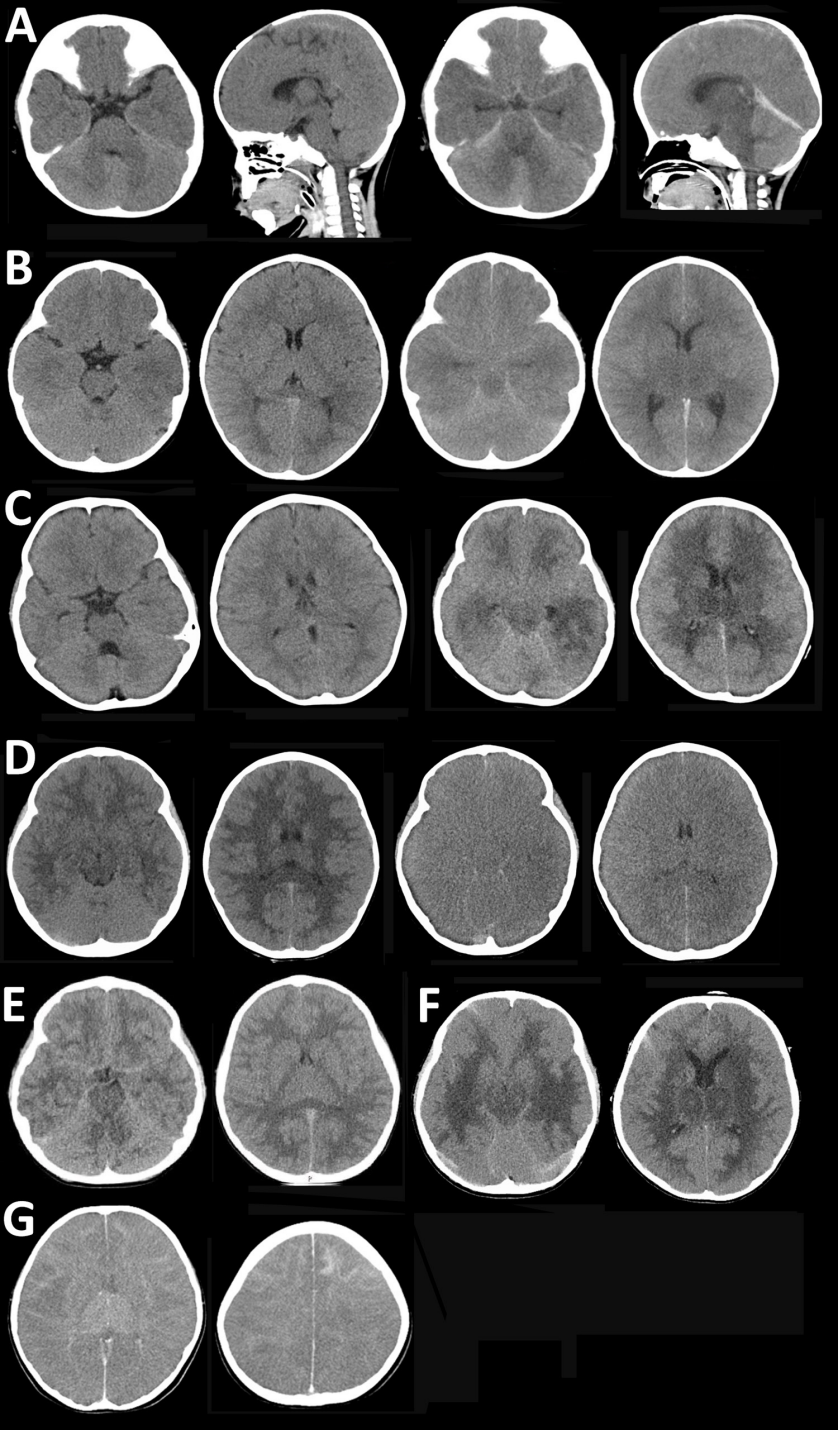
Brain computed tomography imaging for patients with SARS-CoV-2 acute shock with encephalopathy and multiorgan failure/acute fulminant cerebral edema (ASEM/AFCE) for study of life-threatening SARS-CoV-2–associated encephalopathy and multiorgan failure in children, Asia and Oceania, 2022–2024. A) Case-patient 1, showing diffuse cerebral edema (DCE) within 10 hours after onset of neurologic symptoms (left 2 images) and severe DCE and herniation at 18 hours after onset (right 2 images). B) Case-patient 6, showing mild cerebral edema within 4 hours after onset (left 2 images) and severe DCE at 23 hours after onset (right 2 images). C) Case-patient 15, showing DCE 3 hours after onset (left 2 images) and severe DCE with low-density involving the bilateral cerebral white matter and thalamus at 23 hours from the onset (right 2 images). D) Case-patient 19, showing DCE with low-density lesions in the bilateral cerebral white matter within 90 minutes after onset (left 2 images) and severe DCE at 10 hours after onset (right 2 images). E) Case-patient 18, showing mild cerebral edema on day 2 of illness; the patient had no further exacerbation of DCE. F) Case-patent 20, showing DCE with bilateral low-density lesions in the thalamus (left image) and periventricular cerebral white matter within 30 hours after onset (right image). G) Case-patient 23, showing DCE within 7 hours after onset (left image) and a high density of frontal subcortical white matter (right image).

Typical brain imaging findings in ASEM/AFCE (Figure 2) show diffuse cerebral edema (DCE) that appears within 1–24 hours after neurologic onset. In some cases, initial brain imaging demonstrated moderate cerebral edema, but several hours later, severe DCE developed. Some brain images (Figure 2, panel E) were identical to those previously reported (*13*). Typical EEG abnormalities in acute illness included generalized high-amplitude slow waves or diffuse low activity. 

All patients were admitted to the intensive care unit and required tracheal intubation. The duration of hospitalization ranged from 12 hours to 458 days (median 168 hours), and follow-up ranged from 1 to 350 (median 2) days after neurologic onset. The outcomes in our cohort were PCPC 1 in 1 patient (4%), PCPC 5 in 2 patients (8%), and PCPC 6 in 22 patients (88%), including 11 (44% of all 25 patients) who died within 24 hours of hospitalization. Respiratory arrests at <6 hours after neurologic onset occurred in 10 (40%) patients, and cardiac arrests at <6 hours after neurologic onset occurred in 7 (28%) patients (Figure 1).

### Scoping Review

**Figure 3:**
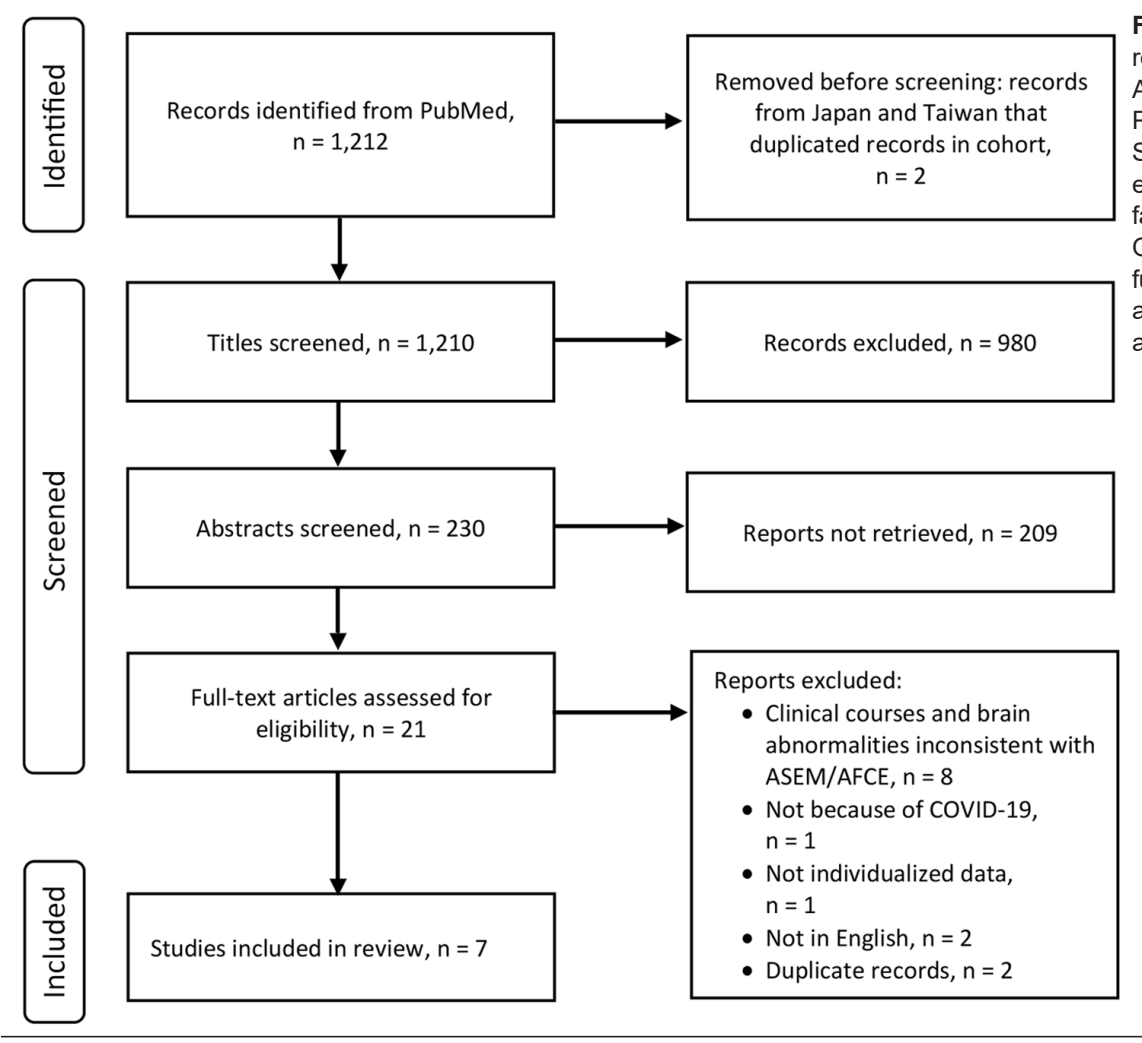
Flow diagram for scoping review of SARS-CoV-2 ASEM/AFCE cases from the literature via PubMed for study of life-threatening SARS-CoV-2–associated encephalopathy and multiorgan failure in children, Asia and Oceania, 2022–2024. AFCE, acute fulminant cerebral edema; ASEM, acute shock with encephalopathy and multiorgan failure.

The scoping review identified 1,212 studies, and we screened 230 relevant articles for eligibility; of those, we included 7 independent studies in the review (*6*,*7*,*14*–*18*) (Figure 3). Of those, 4 case reports and 3 case series involved individualized clinical information of 12 cases and fulfilled the definition of ASEM or AFCE (Table 3). We excluded a case series from Taiwan (*8*) and a case report from Japan (*19*) from the review because they overlapped with our cohort; the duplicates were cases 5, 8, 14, 16, 17, and 25 from Taiwan and case 21 from Japan (Table 1). 

**Table 3:** Cases of SARS-CoV-2 ASE/AFCE reported in the literature from scoping review for study of life-threatening SARS-CoV-2–associated encephalopathy and multiorgan failure in children, Asia and Oceania, 2022–2024*

Country and reference	Patient age	First symptoms with fever	Brain CT/MRI	Outcome by PCPC†	Times from onset to CA
United States (*6*)	7 y	Headaches, abdominal pain, and emesis	DCE	6	NA
United States (7)	<1 y	Gastrointestinal symptoms and status epilepticus	DCE	6	<24 h
	6–12 y	Status epilepticus	DCE and herniation	6	3rd day of admission
	6–12 y	Status epilepticus	DCE and herniation	6	48 h of admission
	3–5 y	Altered awareness, seizure, vomiting, acute respiratory failure, and shock	DCE	NA	NA
United States (*14*)	8 y	Lethargy, myalgias, anorexia, and seizure	DCE	6	NA
Tunisia (*15*)	2 mo	Poor feeding and sleep, seizure, and shock	DCE	6	12 h
India (*16*)	10 y	Repetitive seizures and encephalopathy	DCE and herniation	6	14th day of admission
United States (*17*)	13–17 y	NA	NA	6	NA
	13–17 y	NA	NA	6	NA
South Korea (*18*)	11 y	Status epilepticus	DCE	6	14th day of admission
	9 y	Status epilepticus	DCE	6	NA

*AFCE, acute fulminant cerebral edema; ASEM, acute shock with encephalopathy and multiorgan failure; CA, cardiac arrest; CT, computed tomography; DCE, diffuse cerebral edema; MRI, magnetic resonance imaging; NA, not available; PCPC, pediatric cerebral performance category. †Outcome was graded as follows: presymptomatic state (PCPC = 1), mild disability (PCPC = 2), moderate disability (PCPC = 3), severe disability (PCPC = 4), coma or vegetative state (PCPC = 5), and death (PCPC = 6).

For the remaining cases, age at onset ranged from 2 months to 17 years, including 9 school-aged children. The male:female ratio was 3:1. The reported cases were from India, South Korea, Tunisia, and the United States; however, the ethnicity of each patient was not identified. At least 7 of the 12 patients had status epilepticus as the first neurologic symptom.

### Cytokine/Chemokine Levels in Patients with SARS-CoV-2 ASEM/AFCE

**Figure 4:**
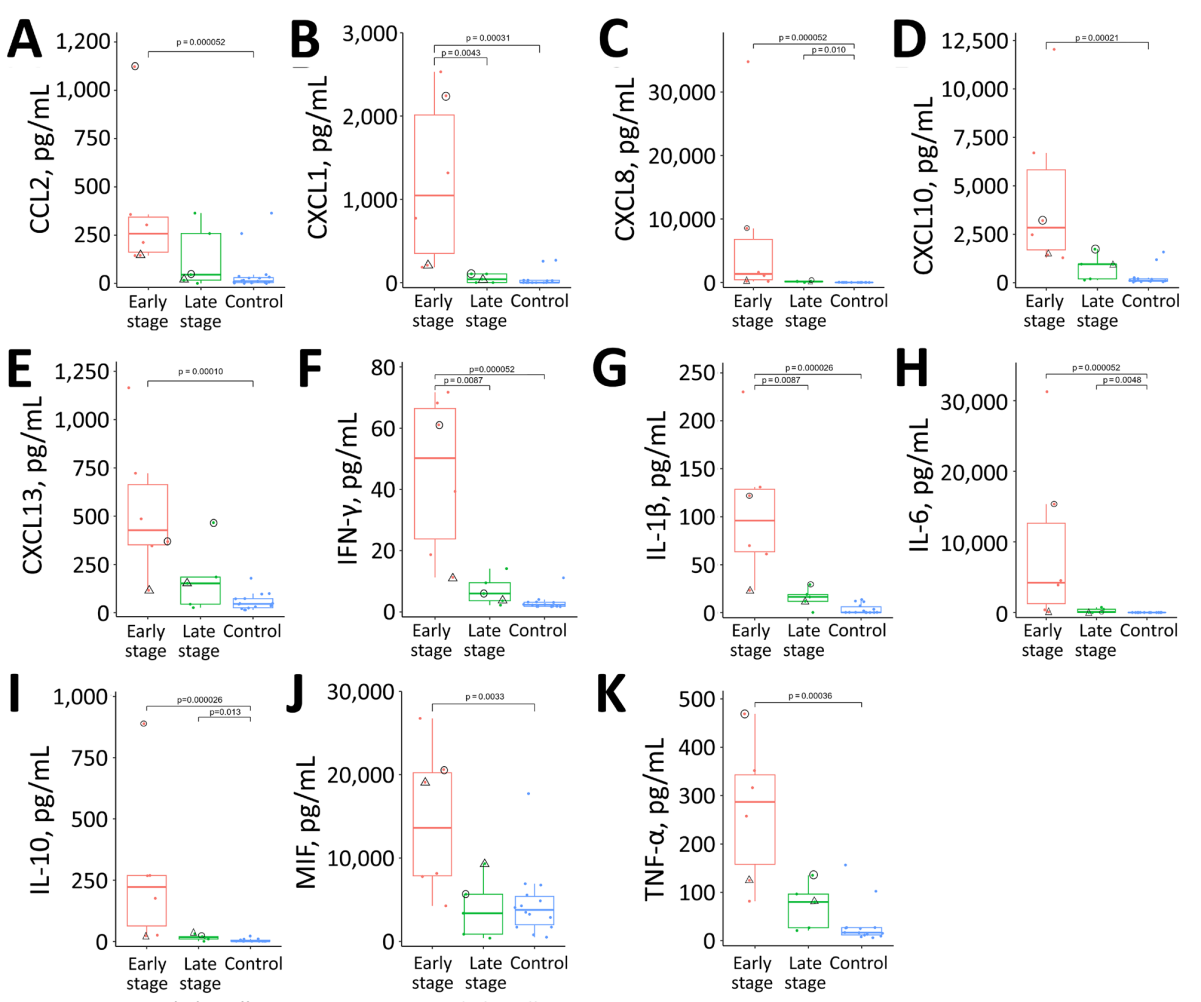
Cytokine and chemokine levels in serum from 9 patients with SARS-CoV-2 acute shock with encephalopathy and multiorgan failure/acute fulminant cerebral edema for study of life-threatening SARS-CoV-2–associated encephalopathy and multiorgan failure in children, Asia and Oceania, 2022–2024. Serum levels were compared among early-stage (red; <24 h after neurologic onset; n = 6), late-stage (green; >24 h after neurologic onset; n = 5), and controls (blue; n = 14). Samples derived from early- and late-stage samples can be compared in 2 cases; circles indicate 1 from case-patient 13 (pediatric cerebral performance category = 6), and triangles indicate 1 from case-patient 18 (pediatric cerebral performance category = 1). Each dot indicates a case-patient; horizontal lines within boxes indicate medians; box tops and bottoms indicate interquartile ranges (IQRs); whiskers indicate 1.5× IQR; outliers are values >1.5× IQR. Kruskal-Wallis p values: A) p = 0.0064; B) p = 0.0019; C) p = 0.00029; D) p = 0.00091; E) p = 0.0018; F) p = 0.00042; G) p = 0.00029; H) p = 0.00025; I) p = 0.00029; J) p = 0.016; K) p = 0.0016. CCL, CC-chemokine ligand; CXCL, CXC-chemokine ligand; IFN, interferon; IL, interleukin; MIF, macrophage migration inhibitory factor; TNF, tumor necrosis factor.

**Figure 5:**
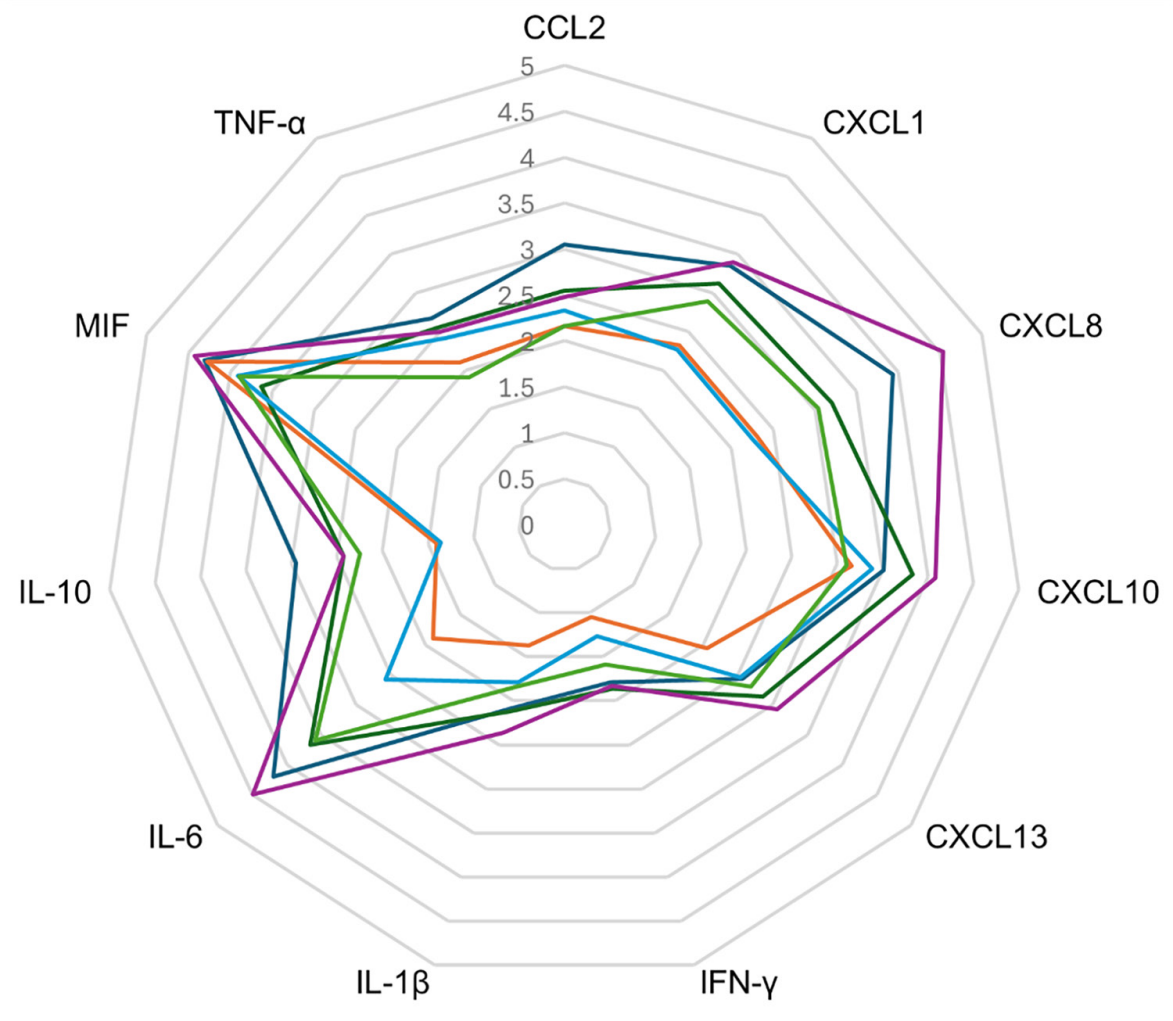
Cytokine and chemokine levels of serum in early illness stage of study of life-threatening SARS-CoV-2–associated encephalopathy and multiorgan failure in children, Asia and Oceania, 2022–2024. Serum levels were compared for 6 patients with early-stage SARS-CoV-2 acute shock with encephalopathy and multiorgan failure/acute fulminant cerebral edema. The values for each cytokine/chemokine are presented on a logarithmic scale; each colored line indicates results for 1 patient. The orange line indicates the case in which the pediatric cerebral performance category = 1. CCL, CC-chemokine ligand; CXCL, CXC-chemokine ligand; IFN, interferon; IL, interleukin; MIF, macrophage migration inhibitory factor; TNF, tumor necrosis factor.

Within the international cohort of SARS-CoV-2 ASEM/AFCE, we determined 11 cytokine/chemokine levels from the 9 case-patients who had given consent to provide their serum. We compared each level among early-stage samples, late-stage samples, and controls (Figure 4). The early-stage samples showed significantly higher levels of all cytokines than did controls. The levels of CXCL8, IL-6, and IL-10 were significantly elevated in late-stage samples compared with those in controls. No cytokines were correlated with outcome, and the case-patient with a low cytokine profile was one of the few survivors (PCPC 1). That case-patient exhibited the lowest values for CXCL13, IFN-γ, IL-1β, IL-10, and IL-6 of all case-patients (Figure 5). When plotting the relationship between serum cytokine and chemokine levels and the interval between neurologic onset and blood collection, the fitted curve suggested that the levels were higher in samples collected earlier, and we observed a peak within 24 hours of onset (Appendix Figure).

## Discussion

A condition in which encephalopathy develops concurrently with or shortly after a febrile infection and does not result from direct invasion of the pathogen into the brain is known as infection-triggered encephalopathy syndrome (ITES) and is distinguished from infectious and autoimmune encephalitis (*3*). ITES is an umbrella term used for several encephalopathy syndromes, each of which has specific clinical and radiologic phenotypes. ASEM and AFCE are the most severe types of ITES syndromes. ASEM is a serious condition in which the patient experiences shock and rapidly progresses to coma and multiorgan failure with progressive diffuse brain edema. ASEM was previously known as hemorrhagic shock encephalopathy syndrome and frequently affects children <1 year of age (*4*,*20*). Conversely, patients with AFCE exhibit encephalopathy and rapidly progressive cerebral edema without shock and multiorgan failure (*5*). Whether ASEM and AFCE are a continuous spectrum of clinical phenotypes with fulminant brain edema remains uncertain.

In this study, we described the clinical manifestations of 25 patients with SARS-CoV-2 ASEM/AFCE from an international cohort. Of note, our SARS-CoV-2 ASEM/AFCE cohort had an older age of onset, with 8 school-aged case-patients out of 25, compared with non–SARS-CoV-2–triggered ASEM, in which onset age has been predominantly in infants and toddlers in previous reports (*4*,*20*,*21*). That result could be attributed to the broad age at onset of SARS-CoV-2 infection. 

All cohort patients with ASEM rapidly progressed to coma, hemodynamic failure, and systemic organ damage in the early stages of onset, similar to results of a previous report on non–SARS-CoV-2–triggered ASEM (*20*–*22*). In addition, more than half of our cohort exhibited shock, multiorgan failure, and DIC within 6 hours of neurologic onset. In contrast, bleeding was evident in less than half (48%) of the 25 cases. Although bleeding has been described as one of the clinical features of hemorrhagic shock encephalopathy syndrome in the past (*4*,*20*), the frequency of bleeding was not high in ASEM, and the absence of bleeding should not rule out a diagnosis of ASEM, nor should bleeding be considered essential for diagnosis. The 88% mortality rate among our patients with SARS-CoV-2 ASEM/AFCE was slightly higher than that reported in a retrospective clinical study of non–SARS-CoV-2–triggered ASEM (*22*). That high mortality rate could be attributed to the high incidence of multiorgan failure in cases of SARS-CoV-2 ASEM. However, because the case series reporting the clinical manifestations of non–SARS-CoV-2 ASEM/AFCE involved a very small population, it remains uncertain whether prognostic differences exist. To address that question, future multicenter collaborative studies and establishment of prospective cohorts will be required for accurate comparisons.

In comparison with our cohort, the scoping review cases exhibited a similarly broad age at onset, from infancy to late childhood, and all cases had a fatal outcome. That finding might indicate a reporting bias toward severe cases in the review. Alternatively, discrepancies between our study cohort and the scoping review might reflect variations in the circulating SARS-CoV-2 strains, which could have contributed to differences in the severity of ASEM/AFCE.

In general, an ITES syndrome can be associated with a range of pathogens, although some viruses have been reported to be associated with specific encephalopathy syndromes (*10*). That fact suggests that not the infection itself but the immunologic responses of the host play a major role in determining the phenotype of ITES. To investigate the immunologic mechanisms, we also measured cytokines/chemokines in the serum samples from patients with of SARS-CoV-2 ASEM/AFCE. When we divided the samples into early-stage and late-stage according to their sampling time, the serum levels of CXCL8, CXCL10, IFN-γ, IL-1β, IL-6, IL-10, and TNF-α were the highest within 24 hours after neurologic onset, which was in agreement with another study that reported serum concentrations of cytokines and chemokines with non–SARS-CoV-2–triggered ASEM (*23*). Within 24 hours of infection, the antigen-nonspecific innate immune response is generally dominant, whereas cellular immunity involving T cells would not be activated that early. In this study, most of the elevated factors were proinflammatory cytokines and chemokines. Innate immune responses are mediated by neutrophils, monocytes, dendritic cells, and natural killer cells. Human CD14^++^CD16^–^ inflammatory monocytes express the chemokine receptor CCR2 and migrate to inflammatory foci via CCL2 (*24*). Such inflammatory monocytes have the ability to produce a broad range of cytokines, including IL-1, IL-6, IL-10, CXCL8, and TNF-α, in response to stimulation (*25*). Of note, the levels of all inflammatory molecules measured in our study were markedly increased. Such a hypercytokine state causes multiorgan failure through the action of IL-1, IL-6, and TNF-α (*26*). Those findings suggest that the high mortality rate associated with SARS-CoV-2 ASEM/AFCE might be related to cytokine storm.

In contrast to the SARS-CoV-2 ASEM/AFCE cases we describe, severe cases of COVID-19 in adults tend to progress to severe acute respiratory distress syndrome and multiorgan failure after 7–10 days of COVID-19 infection (*27*,*28*). In adult cases of COVID-19 with severe acute respiratory distress syndrome and multiorgan failure, increased blood cytokine levels correlated with SARS-CoV-2 detection in the lungs (*29*), indicating that cytokines were produced locally in the lungs. Focusing on cytokine-targeted therapies, tocilizumab, an IL-6–directed therapy, was used in some cases but was not associated with improved prognosis. Rapid progression appears to be less reversible than in other ITES syndromes, such as acute necrotizing encephalopathy. Serum levels of IL-6 were increased in the very early stages of SARS-CoV-2 ASEM/AFCE, but further studies are required to explore the potential benefits of early immunotherapy and other neuroprotective measures.

The first limitation of our study was that we had insufficient clinical information and materials for the analysis because of the small sample size and large number of deaths in our cohort. Also, this multinational cohort has limited generalizability beyond Asia and Oceania. Owing to the retrospective nature of the study, we did not include pediatric cases of SARS-CoV-2 without encephalopathy or non–SARS-CoV-2 ASEM/AFCE. In our review, the details of the treatment and the time course from the onset of neurologic symptoms to death or brain death could not be followed. Age was reported in some cases but not in others. However, this study demonstrated that emerging infections such as SARS-CoV-2 can precipitate devastating ASEM/AFCE that threatens the lives of previously healthy children. Similarly, recent reports of influenza-associated acute necrotizing encephalopathy in the United States during the 2023–2025 influenza seasons have increasingly been documented, underscoring the young age groups affected and the associated high mortality rates (*30*). In view of the serious health effects of severe ITES, particularly ASEM/AFCE, our observations highlight the urgent need to establish international multicenter registries and to implement comprehensive strategies for prevention and surveillance.

In conclusion, our study revealed the clinical features of children with SARS-CoV-2 ASEM/AFCE in patients from Asia and Oceania. Mortality rates are overwhelmingly high because of systemic organ failure, and serum cytokine/chemokine profiles exhibited the presence of hypercytokine states in the early stage of disease onset. Further studies are required to elucidate the mechanisms underlying hypercytokine states and establish treatment strategies. In the meantime, clinicians should be aware of SARS-CoV-2–associated encephalopathy cases such as those we describe and monitor at-risk patients for rapidly progressive brain edema and multiorgan failure.

Appendix Additional information for life-threatening SARS-CoV-2–associated encephalopathy and multiorgan failure in children, Asia and Oceania, 2022–2024.
