# Supplementary material for: Life-Threatening SARS-CoV-2–Associated Encephalopathy and Multiorgan Failure in Children, Asia and Oceania, 2022–2024
Source: Emerg Infect Dis. 2026 Feb;32(2):169–79. doi: 10.3201/eid3202.250549 (PMC12928216; doi:10.3201/eid3202.250549)
Supplement: Appendix — Additional information for life-threatening SARS-CoV-2–associated encephalopathy and multiorgan failure in children, Asia and Oceania, 2022–2024. [file 25-0549-Techapp-s1.pdf]

Article DOI: https://doi.org/10.3201/eid3202.250549

EID cannot ensure accessibility for supplementary materials supplied by authors. Readers who have difficulty accessing supplementary content should contact the authors for assistance.

Life-Threatening SARS-CoV-2–Associated Encephalopathy and Multiorgan Failure in Children, Asia and Oceania, 2022–2024

Appendix

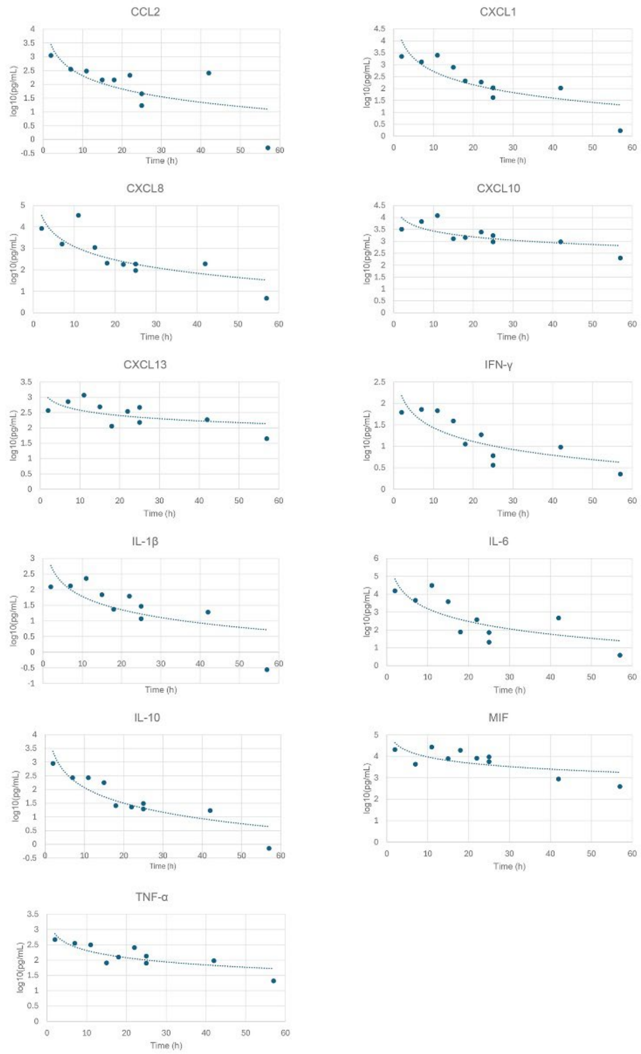

Appendix Figure. The relationship between serum levels of indicated cytokine and chemokine and the interval between the neurologic onset and blood collection. Samples collected more than 72 hours after neurologic onset were excluded. Fitting curves were generated using logarithmic regression in Microsoft Excel. Cytokine and chemokine levels tended to be higher when samples were collected earlier, and the peak occurred within 24 hours after neurological onset. The values for each cytokine/chemokine are presented on a logarithmic scale.
